# Assessment of electrocardiographic markers of acute and long‐term hemodynamic improvement in patients with pulmonary hypertension

**DOI:** 10.1111/anec.12758

**Published:** 2020-04-26

**Authors:** Michał Piłka, Szymon Darocha, Marta Banaszkiewicz, Maria Wieteska‐Miłek, Małgorzata Mańczak, Rafał Mańczak, Piotr Kędzierski, Michał Florczyk, Anna Dobosiewicz, Adam Torbicki, Marcin Kurzyna

**Affiliations:** ^1^ Department of Pulmonary Circulation Thromboembolic Diseases and Cardiology Centre of Postgraduate Medical Education European Health Centre Otwock Poland; ^2^ Department of Gerontology Public Health and Didactics, National Institute of Geriatrics, Rheumatology and Rehabilitation Warsaw Poland

**Keywords:** balloon pulmonary angioplasty, chronic thromboembolic pulmonary hypertension, electrocardiography, pulmonary hypertension, responders

## Abstract

**Background:**

The remodeling of the right heart in patients with chronic pulmonary hypertension (cPH) is associated with the appearance of electrocardiographic (ECG) abnormalities. We investigated the resolution of ECG markers of right ventricular hypertrophy (RVH) caused by acute and long‐term hemodynamic improvement.

**Methods:**

Twenty‐nine (29) patients with chronic thromboembolic pulmonary hypertension (CTEPH) and seven patients with pulmonary arterial hypertension (PAH) were included in the analysis. Patients with CTEPH achieved a significant long‐term hemodynamic improvement following the treatment with balloon pulmonary angioplasty (BPA); all the patients with PAH reported significant acute hemodynamic relief after a single inhalation of iloprost, fulfilling the criteria of responder. Standard 12‐lead ECG was performed before and after intervention.

**Results:**

The interval between baseline and control ECG in CTEPH and PAH groups was 28 (IQR: 17–36) months and 15 min (IQR: 11–17), respectively. Despite similar hemodynamic improvement in both groups, only the CTEPH group presented significant changes in most analyzed ECG parameters: T‐wave axis (*p* = .002), QRS‐wave axis (*p* = .012), P‐wave amplitude (*p* < .001) and duration in II (*p* = .049), R‐wave amplitude in V_1_ (*p* = .017), R:S ratio in V_1_ (*p* = .046), S‐wave amplitude in V_5_ (*p* = .004), R‐wave amplitude in V_5_ (*p* = .044), R:S ratio in V_5_ (*p* = .004), S‐wave amplitude in V_6_ (*p* = .026), R‐wave amplitude in V_6_ (*p* = .01), and R‐wave amplitude in aVR (*p* = .031). In patients with PAH, significant differences were found only for P wave in II (duration: *p* = .035; amplitude: *p* = .043) and QRS axis (*p* = .018).

**Conclusions:**

The effective treatment of cPH ensures improvement in ECG parameters of RVH, but it requires extended time.

## 1 INTRODUCTION

Pulmonary hypertension is a pathologic hemodynamic condition defined as an increased mean pressure in the pulmonary artery ≥25 mm Hg at rest, evaluated via direct measurement during right heart catheterization (RHC), often with the use of Swan‐Ganz catheter (Galie et al., 2015). According to the current classification, chronic pulmonary hypertension (cPH) is divided into five groups, two of which have particularly progressive courses, poor prognosis and require specific therapy (Galie et al., 2015). One of them is pulmonary arterial hypertension (PAH), which is a type of pulmonary hypertension that develops as a result of contraction and gradual obliteration of small pulmonary arterioles (D'Alonzo et al., 1991). The other one is chronic thromboembolic pulmonary hypertension (CTEPH), which is characterized by the presence of chronic thromboembolic lesions in pulmonary arteries that persist despite an effective therapy with antithrombotic medications for at least 3 months (Riedel, Stanek, Widimsky, & Prerovsky, 1982). Both PAH and CTEPH lead to overload, hypertrophy, and dilatation of the right ventricle and finally to its progressive insufficiency and the patient's death. Progression of cardiac remodeling is associated with the appearance of electrocardiographic (ECG) abnormalities described as hypertrophy and overload of the right ventricle (RV). It is not certain which of these abnormalities are associated with acute and with chronic overload of right cardiac cavities. Paradoxically, analysis of the resolution of ECG curve following the use of effective specific therapy may help in discovering the determinants of ECG changes. There are only a few clinical situations enabling the observation of such changes. They include effective balloon pulmonary angioplasty (BPA) in CTEPH, which correspond to model long‐term relief of the RV (Araszkiewicz et al., 2019; Darocha, Kurzyna, Pietura, & Torbicki, 2013; Darocha et al., 2017; Mizoguchi et al., 2012). The different intervention allowing decreasing chronically elevated PAP, but within the minutes, is the administration of vasodilating medications in patients suffering from PAH with retained vasoreactivity of pulmonary vessels, the so‐called “responders” (Rich, Kaufmann, & Levy, 1992). The above‐mentioned term is used to describe the rare group of PAH patients, whose vessels respond by a significant decrease in pulmonary resistance and pressure in pulmonary artery immediately after the administration of vasodilating medication (Galie et al., 2015). In this study, we prospectively observed which ECG abnormalities primarily depend on a sudden decrease in pressure in pulmonary artery, and which abnormalities persist and will most probably require more time for their regression. The above model is a unique phenomenon, which is mainly associated with very rare occurrence of those groups of patients. Currently, there are only a few studies in the literature which analyze ECG curves following the effective treatment of CTEPH with the use of BPA; however, there are no studies on the assessment of ECG markers of improvement after acute vasoreactivity test (Asano et al., 2019; Nishiyama et al., 2018; Pilka et al., 2019; Yokokawa et al., 2019).

## 2 METHODS

Patients hospitalized at the European Health Centre in Otwock in 2013–2018 and diagnosed with de novo PAH and positive pulmonary vessel vasoreactivity (the so‐called “responders”) and patients with CTEPH who were characterized by a significant hemodynamic improvement following the treatment with BPA were included in the prospective analysis. In accordance with the present Guidelines of the European Society of Cardiology, hemodynamic criteria for patient qualification as a “responder” were accepted, that is, a decrease in the mean pulmonary arterial pressure (mPAP) by at least 10 mm Hg to a value below 40 mm Hg with concomitant increase/lack of decrease in cardiac output (CO) (Galie et al., 2015). From the group of patients with CTEPH (*n* = 42), the analysis included those patients (*n* = 29) in whom the mPAP was reduced by at least 10 mm Hg, to a value below 40 mm Hg, as a result of the applied cyclic interventional treatment with BPA. All patients with PAH included in this study were subjected to ECG assessment before and after the administration of inhaled prostacyclin analogue at the dose and manner of inhalation identical to the dose and manner of inhalation during the RHC performed on the previous day, based on which they were qualified as “responders”. ECG and hemodynamic assessment of patients with CTEPH occurred before the start of interventional treatment. Hemodynamic and ECG control of the above‐mentioned group of patients were conducted after at least three sessions of BPA. All the patients expressed their consent to participate in the study. The institutional ethics committee approved the study protocol (decision number 88/PB/2015).

### 2.1 Right heart catheterization and balloon pulmonary angioplasty

The right heart catheterization was conducted, by internal jugular vein or femoral vein approach, using Swan‐Ganz catheter, according to current standards (Kurzyna et al., 2015). Each patient in whom PAH was confirmed by hemodynamic examination, according to the guidelines of the European Society of Cardiology, was subjected to pulmonary vasoreactivity testing using 5 µg of inhaled prostacyclin analogue, iloprost (Galie et al., 2015). Hemodynamic measurements were performed before the vasoreactivity testing and then approximately 15 min after iloprost administration. In the group of CTEPH patients, RHC was conducted directly before the first BPA and then after at least three sessions of BPA. BPA procedures were performed, according to previous protocol (Kurzyna et al., 2017), by two interventional cardiologists experienced in conducting percutaneous vascular procedures. The following hemodynamic parameters were measured or calculated: right atrial pressure (RAP), mPAP and systolic pulmonary arterial pressure (sPAP), pulmonary capillary wedge pressure (PCWP), pulmonary vascular resistance (PVR), cardiac index (CI), stroke volume index (SVI), right ventricular stroke work index (RVSWI), total peripheral resistance (TPR), and CO, which was measured by thermodilution method.

### 2.2 Six‐minute walk test and laboratory tests

A 6‐min walking (6MWT) test was conducted according to the guidelines of the American Thoracic Society (ATS Committee on Proficiency Standards for Clinical Pulmonary Function Laboratories, 2002). The distance achieved in the above‐mentioned test was included in the analysis. In order to assess myocardial necrosis, high sensitivity troponin concentration was assayed using Elecsys Troponin T high sensitive test (Roche; plasma, *N* < 0.003 ng/ml). Biochemical evaluation of the degree of cardiac insufficiency was conducted by the measurement of NTproBNP (N‐terminal pro‐B‐type natriuretic peptide) concentration (Roche; serum, *N* < 125 pg/ml).

### 2.3 Electrocardiogram

**FIGURE 1:**
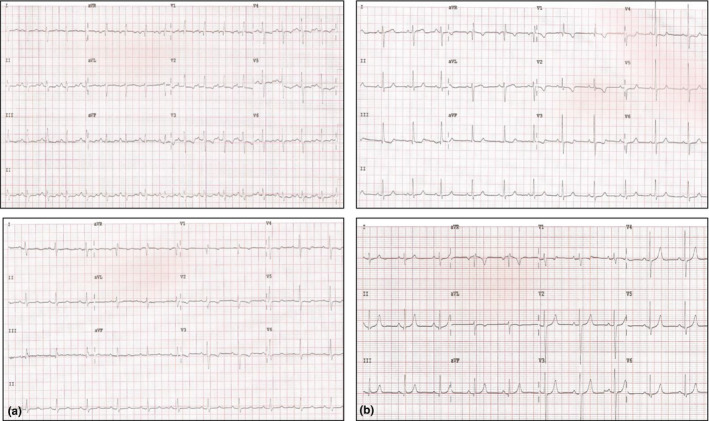
Electrocardiogram (a) before and after iloprost inhalation, (b) before and after three sessions of balloon pulmonary angioplasty

Standard 12‐lead ECG was performed in each patient during calm breathing in horizontal position, using a commercially available ECG device (Philips Page Writer TC 50). The recording paper was moving at the speed of 25 mm/s (1 mV = 10 mm). Two ECG examinations were performed both in PAH and CTEPH patients. For PAH patients, baseline examination was performed 2–3 min before the administration of iloprost inhalation (5 µg) at a dose identical to that during the RHC performed on the previous day; the second examination was conducted approximately 15 min after the administration of the iloprost inhalation mentioned above (when performing this second ECG examination, all the electrodes remained at exactly the same spots on the patient's body as during the examination conducted before iloprost administration; Figure 1). For CTEPH patients, baseline ECG examination was carried out before the initiation of interventional treatment and then together with hemodynamic control after at least 3 BPA sessions (Figure 1). The following ECG characteristics of RV hypertrophy and overload were analyzed: T‐wave axis, QRS‐wave axis, P‐wave amplitude II, P‐wave duration II, R‐wave amplitude V_1_, S‐wave amplitude V_1_, R:S ratio V_1_, S‐wave amplitude V_5_, R‐wave amplitude V_5_, R:S ratio V_5_, S‐wave amplitude V_6_, R‐wave amplitude V_6_, R:S ratio V_6_, RBBB (Right Bundle Branch Block), and R‐wave amplitude aVR.

### 2.4 Statistical analysis

Statistical analyses were performed with STATISTICA PL software (version 13; STATSOFT). Due to the small number of patients in one of the analyzed groups, all the variables were presented as median and interquartile range (IQR). Comparative analyses of the individual groups were conducted using Wilcoxon test. Mann–Whitney *U* test was applied to confirm compliance of baseline hemodynamic parameters with functional parameters in both study groups (CTEPH and PAH) and to compare changes in the values of hemodynamic parameters between the two groups after dedicated specific therapy. McNemar's test was conducted to compare the nominal variables. Spearman's correlation coefficient (for selected parameters Spearman correlation coefficient adjusted for follow‐up time) was calculated to study the correlation between ECG and hemodynamic parameters. In the entire study, the *p*‐value of <.05 was considered statistically significant.

Spearman correlation coefficient adjusted for follow‐up time.

## 3 RESULTS

Twenty‐nine (29) CTEPH patients (aged: 56/IQR:42‐69/), characterized by significant hemodynamic improvement after BPA treatment (median number of BPA sessions 5/IQR 4–6/), and seven PAH patients (aged: 30/IQR: 26–65/), who met the “responder” criteria in the acute vasoreactivity testing, were included in the analysis. The interval between baseline and control ECG in CTEPH and PAH groups was 28 (IQR: 17–36) months and 15 (IQR: 11–17) minutes, respectively.

The PAH patients were younger than the CTEPH patients and had lower mean RAP. Even so, no statistically significant differences were found in the values of hemodynamic and functional parameters for both groups of patients before the application of dedicated intervention (Table 1).

**TABLE 1:** Subject characteristics and comparison of baseline hemodynamic and functional parameters in both groups of patients included in the study (CTEPH and PAH)

Hemodynamic and functional parameters	CTEPH (*n* = 29)	PAH (*n* = 7)	*p*‐Value
RAP (mm Hg)	10 (6–12)	5 (3–8)	**.016**
sPAP (mm Hg)	92 (80–98)	94 (81–101)	.78
mPAP (mm Hg)	52 (48–57)	58 (49–63)	.39
PVR (Wood units)	10.02 (7.96–12.94)	12.01 (7.87–12.71)	.749
TPR (Wood units)	12.01 (10.08–15.78)	13.10 (9.48–14.19)	1.000
CI (L min^−1^ m^−2^)	2.33 (2.03–2.78)	2.68 (2.60–2.77)	.401
SVI (ml/m^2^)	33.69 (25.59–38.32)	37.21 (27.11–41.20)	.472
RVSWI (g m m^−2^ beat^−1^)	20.19 (14.20–23.45)	20.64 (17.78–33.62)	.215
NTproBNP (pg/ml)	991 (449–2,110)	414 (165–932)	.119
Troponin (pg/ml)	0.008 (0.006–0.014)	0.006 (0.003–0.01)	.156
6MWT (m)	370 (222–471)	468 (291–588)	.271
WHO—functional class, *n*	3 (3–3)	3 (2–3)	.208

Note

The data are presented as median (IQR).

Abbreviations: 6MWT, 6‐min walk test; CI, cardiac index; CTEPH, chronic thromboembolic pulmonary hypertension; mPAP, mean pulmonary arterial pressure; NTproBNP—N‐terminal pro‐B‐type natriuretic peptide; PAH, pulmonary arterial hypertension; PVR, pulmonary vascular resistance; RAP, right atrial pressure; RVSWI, right ventricular stroke work index; sPAP, systolic pulmonary arterial pressure; SVI, stroke volume index; TPR, total pulmonary resistance; WHO FC, World Health Organization Functional Class. Statistically significant *p* values (*p* < .05) are indicated in bold.

**FIGURE 2:**
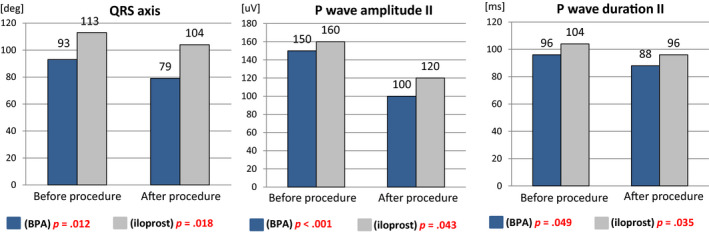
Comparison of electrocardiographic parameters in the individual groups of patients before and after the application of the appropriate specific therapy (BPA for CTEPH/iloprost inhalation for PAH)

In CTEPH patients subjected to BPA procedure, significant statistical improvement was achieved in most analyzed electrocardiographic parameters. In contrast, in PAH patients subjected to iloprost inhalation, significant differences were demonstrated only for P wave in II and QRS axis (Table 2; Figure 2).

**TABLE 2:** Comparison of hemodynamic and electrocardiographic parameters in the individual groups of patients before and after the appropriate intervention (BPA for CTEPH/iloprost inhalation for PAH)

	CTEPH (before intervention)	CTEPH (after intervention)	*p*‐Value	PAH (before intervention)	PAH (after intervention)	*p*‐Value
Hemodynamic parameters						
RAP (mm Hg)	10 (6–12)	7 (6–9)	**<.001**	5 (3–8)	4 (3–5)	.144
sPAP (mm Hg)	92 (80–98)	41 (39–54)	**<.001**	94 (81–101)	53 (40–59)	**.018**
mPAP (mm Hg)	52 (48–57)	25 (22–31)	**<.001**	58 (49–63)	35 (25–36)	**.018**
PVR (Wood units)	10.02 (7.96–12.94)	3.55 (2.60–4.52)	**<.001**	12.01 (7.87–12.71)	4.18 (3.37–6.03)	**.018**
TPR (Wood units)	12.01 (10.08–15.78)	5.50 (4.37–6.53)	**<.001**	13.10 (9.48–14.19)	6.92 (4.78–7.40)	**.018**
CI (L min^−1^ m^−2^)	2.33 (2.03 – 2.78)	2.77 (2.50–3.20)	**.016**	2.68 (2.60–2.77)	2.97 (2.69–3.16)	**.018**
SVI (ml/m^2^)	33.69 (25.59 – 38.32)	40.77 (33.87–47.69)	**<.001**	37.21 (27.11–41.20)	45.80 (31.46–56.09)	**.018**
RVSWI (g m m^−2^ beat^−1^)	20.19 (14.20 –23.45)	12.45 (9.76–14.07)	**<.001**	20.64 (17.78–33.62)	18.48 (11.26–19.94)	**.028**
Electrocardiographic parameters						
T‐wave axis (degrees)	−1.5 (−39.5–29.5)	30 (1–47)	**<.001**	1 (−25–24)	5 (1–15)	.176
QRS‐wave axis (degrees)	93 (62.5–130)	79 (39–93)	**.012**	113 (88–118)	104 (66–113)	**.018**
P‐wave amplitude II (µV)	150 (100–200)	100 (100–120)	**<.001**	160 (140–180)	120 (100–140)	**.043**
P‐wave duration II	96 (80–108)	88 (72–104)	**.049**	104 (88–112)	96 (64–104)	**.035**
R‐wave amplitude V_1_ (µV)	240 (100–460)	120 (100–240)	**.017**	400 (380–880)	600 (180–880)	.237
S‐wave amplitude V_1_ (µV)	290 (170–510)	400 (260–580)	.175	200 (100–660)	280 (100–1,200)	.225
R:S ratio V_1_	0.54 (0.31–1.15)	0.38 (0.17–0.60)	**.046**	1.35 (0.58–2.93)	0.80 (0.57–3.14)	.917
S‐wave amplitude V_5_ (µV)	660 (470–940)	440 (380–660)	**.004**	680 (380–780)	440 (100–860)	.398
R‐wave amplitude V_5_ (µV)	860 (660–1,190)	1,040 (820–1,240)	**.044**	900 (700–1,300)	720 (200–1,560)	.345
R:S ratio V_5_	1.19 (0.87–2.15)	2.25 (1.71–2.95)	**.004**	1.24 (1.15–2.93)	1.85 (1.17–2.25)	.49
S‐wave amplitude V_6_ (µV)	400 (240–540)	300 (140–460)	**.026**	300 (240–600)	380 (80–500)	.345
R‐wave amplitude V_6_ (µV)	720 (530–1,070)	920 (660–1,100)	**.01**	540 (480–740)	540 (140–740)	.345
R:S ratio V_6_	1.97 (1.16–2.94)	2.62 (1.80–5.27)	.096	1.6 (1.23–3.47)	1.75 (1.26–4.63)	.499
RBBB (Right Bundle Branch Block)	4 (13.8%)	3 (10.3%)	1.0000	0	0	—
R‐wave amplitude (aVR)	40 (20–230)	20 (20–40)	**.031**	360 (80–540)	160 (60–500)	.237

Note

The data are presented as median (IQR) or number (%).

Abbreviations: CI, cardiac index; mPAP, mean pulmonary arterial pressure; PVR, pulmonary vascular resistance; RAP, right atrial pressure; RVSWI, right ventricular stroke work index; sPAP, systolic pulmonary arterial pressure; SVI, stroke volume index; TPR, total pulmonary resistance. Statistically significant *p* values (*p* < .05) are indicated in bold.

Table 3 illustrates the comparison of the changes in the values of hemodynamic parameters between both study groups after dedicated intervention.

**TABLE 3:** Comparison of changes in the values of hemodynamic parameters between both study groups after dedicated intervention

Hemodynamic parameters	CTEPH (∆)	PAH (∆)	*p*‐Value
RAP (mm Hg)	5 (2–7)	0 (0–5)	**.024**
sPAP (mm Hg)	45 (37–50)	41 (28–55)	.589
mPAP (mm Hg)	27 (22–30)	25 (18–33)	.764
PVR (Wood units)	6.28 (3.38–9.05)	5.67 (4.32–9.08)	1.000
TPR (Wood units)	6.73 (4.15–9.06)	5.20 (3.92–7.62)	.749
CI (L min^−1^/m^−2^)	0.31 (0.06–0.66)	0.28 (0.17–0.47)	.719
SVI (ml/m^2^)	8.62 (0.46–12.97)	11.05 (7.23–14.89)	.603
RVSWI (g m m^−2^ beat^−1^)	6.46 (2.53–10.05)	7.06 (3.34–8.06)	.873

Note

The data are presented as median (IQR).

Abbreviations: **∆,** change in the value (post–pre); CI, cardiac index; CTEPH, chronic thromboembolic pulmonary hypertension; mPAP, mean pulmonary arterial pressure; PAH, pulmonary arterial hypertension; PVR, pulmonary vascular resistance; RAP, right atrial pressure; RVSWI, right ventricular stroke work index; sPAP, systolic pulmonary arterial pressure; SVI, stroke volume index; TPR, total pulmonary resistance. Statistically significant *p* values (*p* < .05) are indicated in bold.

**FIGURE 3:**
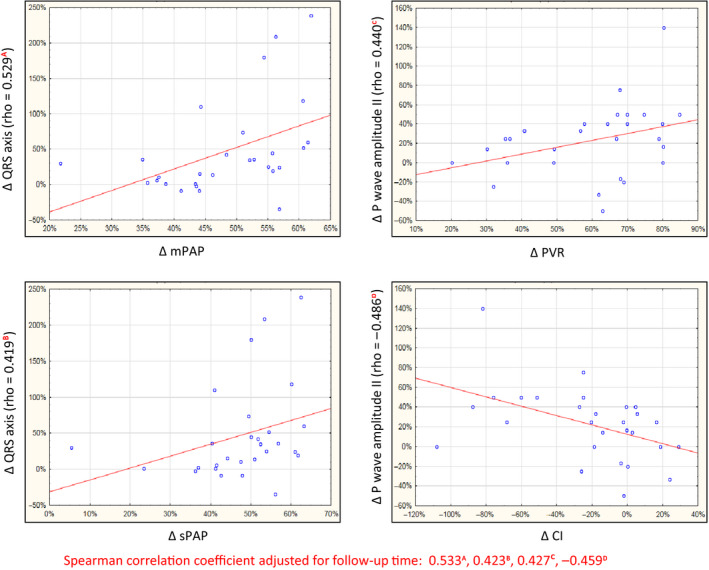
Selected correlations in percentage change in the values of the electrocardiographic and hemodynamic parameters in patients with chronic hemodynamic improvement (CTEPH)

For CTEPH patients who achieved long‐term hemodynamic improvement, statistically significant correlations (*p* < .05) were obtained in terms of percentage change in the mPAP and percentage change in the values of the following ECG parameters: QRS axis (rho = 0.529), S‐wave amplitude V_5_ (rho = 0.634), R:S ratio V_5_ (rho = −0.638), and S‐wave amplitude V_6_ (rho = −0.638). In relation to PVR, the same correlation was observed for the change in T axis (rho = 0.472), QRS axis (rho = 0.607), P‐wave amplitude II (rho = 0.440), S‐wave amplitude V_5_ (rho = 0.572), R:S ratio V_5_ (rho = −0.65), and S‐wave amplitude V_6_ (rho = −0.565). However, the percentage change in sPAP was significantly correlated with the percent change in the following ECG parameters: QRS axis (rho = 0.419), S‐wave amplitude V_5_ (rho = 0.597), R:S ratio V_5_ (rho = −0.555), and S‐wave amplitude V_6_ (rho = −0.555). In addition, during chronic hemodynamic improvement, the percentage change in QRS axis was significantly correlated with the percent change in the values of the following hemodynamic parameters: TPR (rho = 0.689), CI (rho = −0.486), and SVI (rho = −0.578). Statistically significant correlation was also obtained for the percent change in the value of the P‐wave amplitude and percentage change in the following hemodynamic parameters: TPR (rho = 0.460), CI (rho = −0.473), and SVI (rho = −0.430). Selected correlations after adjusting for follow‐up time are presented in Figure 3. In case of acute hemodynamic improvement, no statistically significant correlation between the changes in percentage values of the individual hemodynamic and ECG parameters was obtained (*p* > .05).

## 4 DISCUSSION

This study attempts, for the first time, to show which of the ECG parameters resolve in the situation of acute RV relief measured in minutes and which ones during long‐term hemodynamic improvement measured in months in patients with cPH.

Both PAH and CTEPH lead to overload, hypertrophy, RV dilatation and progressive insufficiency, and patient's death. Progression of heart failure is associated with the appearance of ECG abnormalities.

Long‐term retaining RV relief leads to a significant improvement in its function, thus boosting the chances of survival of cPH patients (Rich et al., 1992). Knowledge of the relationship between the specific ECG parameters of hypertrophy and overload of right cardiac cavities with acute and long‐term hemodynamic improvement in the selected groups of patients with pulmonary hypertension may be useful for monitoring the effectiveness of the applied therapy and may have a prognostic value.

In patients with PAH, diagnostic values of the selected ECG parameters—such as presence of qR complex in V_1_, P‐wave amplitude in II, P‐wave duration, QRS axis, QRS duration, R‐ and S‐wave amplitude in V_5_ and V_6_, and R‐ and S‐wave amplitudes in V_1_—were confirmed in several studies (Al‐Naamani et al., 2008; Bonderman et al., 2011; Kanemoto, 1981; Kopec et al., 2012; Lehtonen, Sutinen, Ikaheimo, & Paakko, 1988; Sun et al., 2012; Tonelli, Baumgartner, Alkukhun, Minai, & Dweik, 2014; Waligora et al., 2017).

One of the few studies assessing the correlation between the selected ECG and hemodynamic parameters, before the introduction of pharmacotherapy targeted at pulmonary arterioles, was published by Kanemato et al. In that study, sPAP showed a significant correlation with the R‐wave amplitude and R:S‐wave amplitude ratio in V_1_. Moreover, the R‐wave amplitude in V_1_ above 1.2 mV enabled the identification, with high sensitivity (94%) and specificity (47%), of patients with sPAP above 90 mm Hg. The study also demonstrated a significant correlation between CI and the following ECG parameters: R‐wave amplitude in V_5_ and V_6_, R/S ratio in V_5_ and V_6_ (Kanemoto, 1988). Our considerations did not reveal any statistical significance in relation to the change in R‐wave amplitude in V_1_ during acute vasoreactivity testing, which is in contrast to patients with long‐term hemodynamic improvement, in whom statistical significance of the mentioned ECG parameter was demonstrated. In our study, a change in the value of R‐wave amplitude in V_1_ was not significantly correlated with a change in the value of any of the hemodynamic parameters (CTEPH and PAH patients).

Our study demonstrated significant changes in R‐ and S‐wave amplitude in V_5_ and V_6_ values but only in the model of long‐term hemodynamic improvement, as in previous studies on significant hemodynamic improvement in CTEPH patients who received interventional therapy (Nishiyama et al., 2018; Pilka et al., 2019). The value of the T‐wave axis in relation to the long‐term hemodynamic improvement model in the current study was the same as in a recent publication by us (Pilka et al., 2019). For RBBB, none of the patients from the PAH group met the criteria for the diagnosis, and the differences in its frequencies before and after the treatment with BPA in CTEPH patients were not statistically significant.

Several previous studies, for example, by Waligóra et al. and Cheng et al., demonstrated the importance of the P‐wave amplitude, when compared to the other ECG parameters, in revealing hemodynamic improvement in patients with PAH, following the application of targeted pharmacotherapy (Cheng et al., 2017; Waligora, Tyrka, Podolec, & Kopec, 2018). The study by Cheng et al. showed a significant correlation between the P‐wave amplitude and the mPAP value (*r* = .35, *p* < .001), and between the P‐wave amplitude and CI (*r* = −.22, *p* = .002). The value of the P‐wave amplitude and its significance were also confirmed in CTEPH patients subjected to pulmonary endarterectomy. The reduction in the P‐wave amplitude was correlated with a reduction in PVR and mPAP; the P‐wave amplitude was one of the few electrocardiographic parameters correlated with PVR and mPAP. P‐wave amplitude, R‐wave amplitude in V_1_, and the number of patients with negative T wave in V_1_–V_3_ decreased significantly, with rapid improvement in right heart hemodynamics, 1 month after surgery, without any further change in 1 year (Ghio et al., 2016). In our study, the change in the P‐wave amplitude in II, in the long‐term model, is significantly correlated with changes in the PVR (rho = 0.44), CI (rh = −0.473), TPR (rho = 0.46), and SVI (rho = −0.43) values. An extremely interesting observation is the fact that a change in morphology and amplitude of the P wave in II reflects acute hemodynamic improvement (P‐wave amplitude is one of only two ECG variables capable of such reflection), following the application of a vasodilating medication.

For the QRS complex, the value of the change in the axis was confirmed in patients with CTEPH after the application of hemodynamically effective interventional therapy (Nishiyama et al., 2018). Similarly, the duration of the QRS complex concerning the prognosis was well‐documented in the CTEPH patients (Asano et al., 2019). In our study, the QRS complex axis is the second variable whose value changed significantly not only in the long‐term model, but also during the acute hemodynamic improvement.

A study, by Henkens et al., of 81 patients, which assessed the usefulness of electrocardiography in monitoring the effectiveness of the treatment of pulmonary arterial hypertension, proved the significant usefulness of a change in the P‐wave amplitude (area under the curve [AUC], 0.80; 95% CI, 0.82 to 0.97; *p* < .001) and the QRS complex axis (AUC, 0.70; 95%, 0.52–0.89; *p* = .03) in the assessment of hemodynamic response, following the application of specific therapy (Henkens et al., 2008).

To date, none of the ECG studies on pulmonary hypertension has been able to demonstrate which of the parameters of right cardiac cavity overload respond quickly to acute relief of the right heart and which require persistent hemodynamic improvement. In this regard, our model for analysis of the respondents is unique. Taking into consideration the results of our study, it appears that the P wave and the QRS complex axis are the most sensitive parameters for acute/rapid hemodynamic improvement in patients with pulmonary hypertension.

To conclude, in patients with cPH, regardless of the therapy used, ECG improvement requires extended time. Among the recognized ECG parameters of RV hypertrophy/overload, only the QRS complex axis and the P‐wave morphology (amplitude and duration) respond significantly to a sudden decrease in pulmonary arterial pressure. A cheap and generally available ECG examination, in the case of long‐term hemodynamic improvement in patients with CTEPH, may become a simple tool for monitoring the effectiveness of interventional therapy in time and may help in making decision on its termination. Perhaps, the severity of the change in QRS complex axis and P‐wave morphology (amplitude and duration) in II will be clinically significant in making therapeutic decisions and identifying long‐term responders. However, further observations and research on a larger group of patients are necessary.

### 4.1 Limitations

The main limitation of the study is the small group of patients, especially the PAH patients who tested positive to vasoreactivity. However, it should be noted that this is an extremely rare group of patients, whose prospective observation over time is extremely difficult.

The second limitation is the lack of analysis of the change in morphology and function of the right cardiac cavities assessed by diagnostic imaging (echocardiography, cardiac NMR), in the context of ECG changes.

## 5 CONCLUSION

In patients with cPH, ECG improvement requires time. Electrocardiographic monitoring of the effectiveness of the applied therapy is particularly valuable in the group of patients with pulmonary hypertension with significant and long‐term hemodynamic improvement. Electrocardiogram responds only slightly to acute hemodynamic improvement; however, the analysis of these slight changes in the selected ECG parameters may have clinical implications.

## CONFLICT OF INTEREST

Michał Piłka and Michał Florczyk have each received personal fees from Actelion, MSD, and AOP Orphan. Szymon Darocha and Adam Torbicki have each received grants and personal fees from Actelion, MSD, Bayer, and AOP Orphan. Marcin Kurzyna has received grants and personal fees from Actelion, MSD, Bayer, Biotronik and AOP Orphan. The following authors report no conflicts of interest: Marta Banaszkiewicz, Małgorzata Mańczak, Rafał Mańczak, Piotr Kędzierski, and Anna Dobosiewicz.

## AUTHOR CONTRIBUTIONS

MP, AT, MK substantially contributed to conception and design. MP, MB, MW, RM, PK, MF, AD contributed acquisition of data. MP, MM, and MK analyzed the data. All authors were involved in drafting the manuscript or revising it.

## ETHICAL APPROVAL

The institutional ethics committee approved the study protocol, and informed consent was obtained from each patient before study (decision number 88/PB/2015).
